# Translational cognitive systems: focus on attention

**DOI:** 10.1042/ETLS20220009

**Published:** 2022-11-21

**Authors:** Benjamin Z. Roberts, Jared W. Young

**Affiliations:** 1 Department of Psychiatry, University of California, San Diego, 9500 Gilman Drive, La Jolla, CA 92093, U.S.A.; 2 Research Service MIRECC, VISN 22, Veterans Affairs San Diego Healthcare System, San Diego, CA 92161, U.S.A.

**Keywords:** attention, continuous performance test, cross-species, translational, vigilance

## Abstract

Cognitive dysfunction, particularly attentional impairment, is a core feature of many psychiatric disorders, yet is inadequately addressed by current treatments. Development of targeted therapeutics for the remediation of attentional deficits requires knowledge of underlying neurocircuit, cellular, and molecular mechanisms that cannot be directly assayed in the clinic. This level of detail can only be acquired by testing animals in cross-species translatable attentional paradigms, in combination with preclinical neuroscience techniques. The 5-choice continuous performance test (5C-CPT) and rodent continuous performance test (rCPT) represent the current state of the art of preclinical assessment of the most commonly studied subtype of attention: sustained attention, or vigilance. These tasks present animals with continuous streams of target stimuli to which they must respond (attention), in addition to non-target stimuli from which they must withhold responses (behavioral inhibition). The 5C-CPT and rCPT utilize the same measures as gold-standard clinical continuous performance tests and predict clinical efficacy of known pro-attentional drugs. They also engage common brain regions across species, although efforts to definitively establish neurophysiological construct validity are ongoing. The validity of these tasks as translational vigilance assessments enables their use in characterizing the neuropathology underlying attentional deficits of animal models of psychiatric disease, and in determining therapeutic potential of drugs ahead of clinical testing. Here, we briefly review the development and validation of such tests of attentional functioning, as well as the data they have generated pertaining to inattention, disinhibition, and impulsivity in psychiatric disorders.

## Introduction

Psychiatric disorders affect roughly one in every four people worldwide. Cognitive system dysfunction is a critical factor across diseases, either defining a given condition or closely correlating with functional outcome. Impaired attention is common across many such disorders, including attention deficit hyperactivity disorder (ADHD), schizophrenia, and bipolar disorder (BD) [1,2]. The lack of treatments available for the specific remediation of attentional dysfunction in schizophrenia and BD constitutes a ‘great unmet therapeutic need' [3], thus making the characterization of underlying neural mechanisms vitally important. While functional magnetic resonance imaging (fMRI) and electroencephalographic (EEG) recording in humans identifies important brain regions and networks, function at the neuronal, neurotransmitter, and receptor levels can only be elucidated by animal testing [4]. Here, we describe and comment on the development and findings of the 5-choice continuous performance test (5C-CPT) and rodent continuous performance test (rCPT), cross-species translatable tasks that enable such high-resolution study of the most commonly assessed subtype of attention: sustained attention or vigilance, the attendance to one or more discrete stimuli for extended periods of time [5].

## Early tests of attentional functioning in rodents

**Figure 1.**
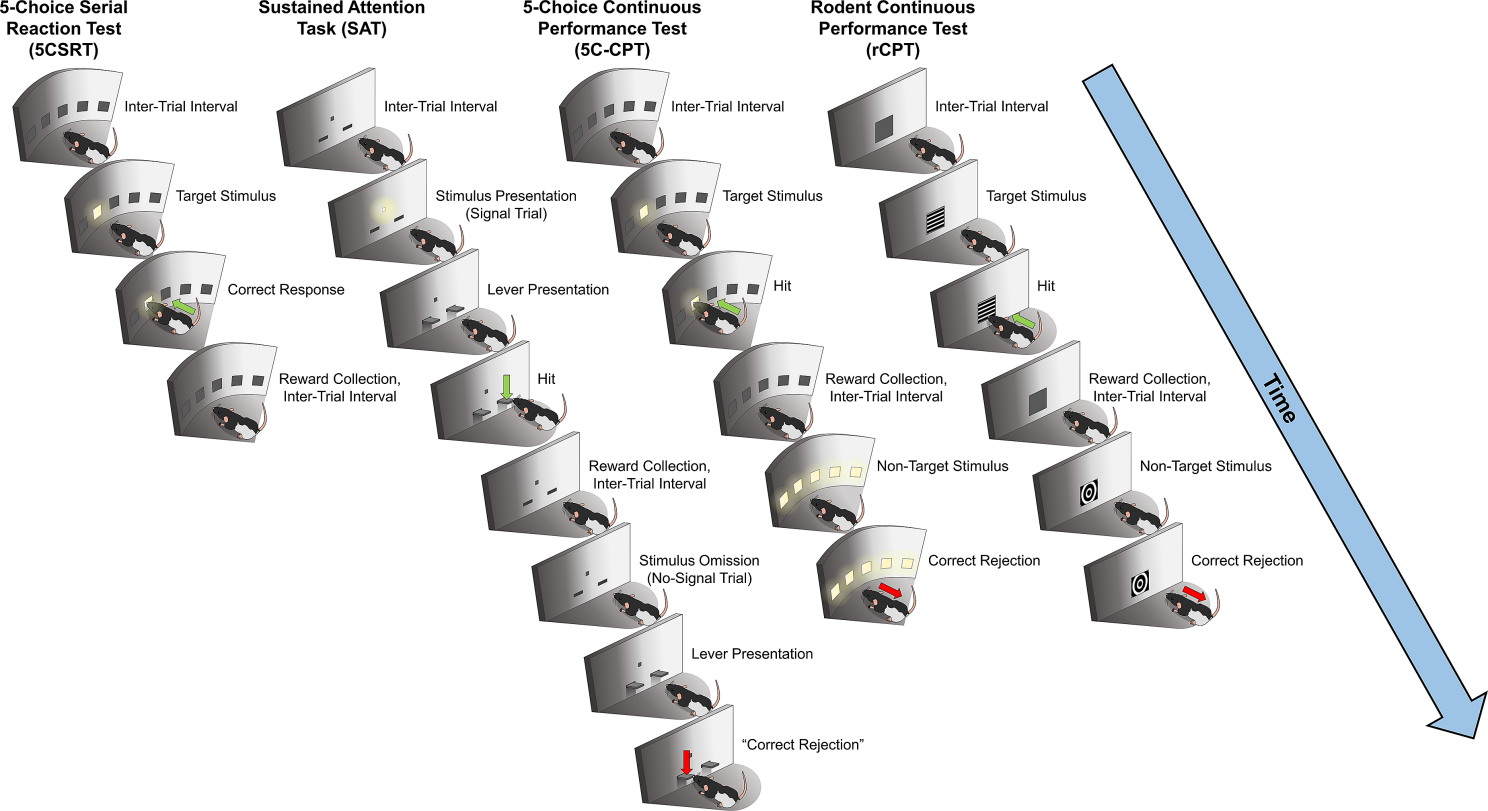
Schematics of common rodent vigilance tasks. The 5-choice serial reaction test (5CSRT; far left) requires rodents to attend to a panel of five horizontally arranged apertures and nosepoke in whichever one illuminates. Task performance is gauged by number of correct versus incorrect localizations of stimuli across trials. In the sustained attention task (SAT; middle left), rodents monitor a single location for the appearance of a stimulus light. Two seconds after delivery (signal trial) or omission (no-signal trial) of the stimulus, rodents are presented with two levers; rodents must then press one lever if the stimulus light had been activated, or the other lever if the stimulus had been absent. Correct responses are termed ‘hits' in signal trials and ‘correct rejections' in no-signal trials. Incorrect responses are termed ‘misses' and ‘false alarms' in signal and no-signal trials, respectively. The 5-choice continuous performance test (5C-CPT; middle right) utilizes the same array of five horizontally arranged stimulus presentation/response apertures as the 5CSRT, but differs from this earlier task by including relatively infrequent ‘non-target stimuli' — simultaneous illuminations of all five apertures, which require rodents to refrain from responding. Correct and incorrect localizations of target stimuli are recorded as hits and incorrects, respectively, with failures to respond at all to target stimuli logged as misses. Responses to non-target stimuli are recorded as false alarms, and appropriately withheld responses to non-target stimuli are recorded as correct rejections. The rodent continuous performance test (rCPT; far right) delivers stimuli and receives responses via a single central touchscreen. The rCPT utilizes the same trial types and outcome measures as the 5C-CPT, but delivers nuanced non-spatial patterns as stimuli instead of spatially distributed lights. Target and non-target rCPT stimuli depicted in the figure were taken directly from Kim et al. [80].

Numerous tests have been developed to assess attention in rodents over the last four decades [4,6,7]. The 5-choice serial reaction task (5CSRT; Figure 1), first adapted by Robbins and colleagues from the Leonard's 5CSRT used in clinical research [8], requires rats [9] and mice [10] to nosepoke wherever a cue light briefly appears in one of five spatial locations. Task accuracy is determined from correct versus incorrect localizations of the cue light, while motoric impulsivity is reported by premature responses (i.e. nosepokes in unlit apertures prior to stimulus presentation). Traditional human attentional testing requires more than continual responding to targets, however. As early as the first formal assessment of vigilance in 1950 [11], human attention paradigms have incorporated non-target stimuli from which subjects must withhold responses while continuing to respond to target stimuli. Such paradigms came to be called continuous performance tests (CPTs) [12]. Non-target trials enable the use of signal detection theory (SDT) to quantify sensitivity to signal versus noise stimuli [13,14]. The absence of non-target stimuli from the 5CSRT therefore hinders direct cross-species comparison with clinical CPTs via SDT, likely leading Robbins to conclude that the task's ‘requirements fall short of that which is normally regarded as vigilance' [15]. Nevertheless, the 5CSRT became one of the most common methods of assessing sustained attention in rodents worldwide, despite its official specification as solely a test of reaction time [16]. Indeed, the greater relevance of the task to this latter domain than the former is illustrated by findings of reaction time deficits in schizophrenia patients assessed in a reverse-translated version of the 5CSRT, but no alterations in attentional measures (corrects versus incorrects, or omitted responses) [17]. Therefore, while the 5CSRT certainly assays aspects of sustained attention, both its translatability to clinical attention/vigilance measures and its sensitivity in detecting psychopathology-associated attentional deficits have been questioned [18].

This limitation of the 5CSRT was partly addressed by the Sustained Attention Task (SAT; Figure 1; [19]). Based on a task designed by Bushnell et al. [20], the SAT requires rats to monitor a single location for the appearance of a 25–500 ms cue stimulus, then press one response lever (presented 2 sec later) if the stimulus appeared, and another if it was absent. The incorporation of a no-signal condition ostensibly generates ‘non-target' behavioral measures (‘correct rejections' of, and ‘false alarms' to, no-signal trials) in addition to the ‘target' measures collected by the earlier 5CSRT (corrects and incorrects). A reverse-translated version of the SAT revealed similar sensitivity to distractors and changes in signal strength across rats and humans [21], and — unlike the 5CSRT — identified attention-specific performance deficits in people with schizophrenia [22]. Despite this improved clinical relevance and addition of a no-signal condition, however, the SAT still critically differs from gold-standard human testing paradigms (e.g. CPTs) as it does not assess response inhibition. Active responses (lever-presses) are required regardless of whether a stimulus is presented or withheld, and no distinct non-target stimuli are delivered. Correct rejections and false alarms in the SAT therefore differ from 5CSRT measures only in terms of trial type (i.e. signal versus no-signal). Furthermore, the 2 sec interval between stimulus delivery/omission and response lever presentation in the SAT potentially entangles attention with memory (this issue is largely mitigated in the human task, however; [21]). Hence, while the SAT and earlier 5CSRT have generated a considerable corpus of rodent attentional literature, the validity of these tasks as assessments of sustained attention in the context of clinical measures remains debatable.

## Development and validation of the 5-choice continuous performance test (5C-CPT)

The need for rodent vigilance tests that also assess response inhibition led to the development of the 5C-CPT (Figure 1; [18]). Like the 5CSRT, the 5C-CPT requires animals to respond in individually illuminated holes arrayed across a panel (target stimuli), but with the added criterion that they inhibit responding when all five holes are lit simultaneously (non-target stimuli). Thus, as in human CPTs, both hit rate (corrects or ‘hits' versus omitted responses or ‘misses') and false alarm rate (false alarms versus correct rejections) can be calculated, with SDT enabling measurements of response ‘bias’ (likelihood of responding) and overall vigilance. These measures also enable quantification of rodent response strategies in a manner similar to clinical CPTs; i.e. individuals may demonstrate strategies that are relatively liberal or conservative depending on the degree to which they prioritize maximization of hit rate over non-target response inhibition or vice versa [23–25]. Furthermore, whereas SAT sessions comprise equal numbers of signal and no-signal trials, the 5C-CPT presents target and non-target stimuli in a 5 : 1 ratio [18]. By delivering only occasional non-target stimuli within a stream of mainly targets, the 5C-CPT promotes development of a dominant (i.e. prepotent; [26]) target response that is difficult to suppress upon the eventual presentation of a non-target stimulus, thereby enabling assessment of cognitive control [27]. Importantly, the 5C-CPT has since been reverse-translated for use in humans in a manner that enables concurrent EEG recording [28] and fMRI [29], and that is sensitive to attentional deficits in psychiatric populations [30,31].

In addition to the face validity afforded by these cross-species attentional measures, the 5C-CPT also exhibits construct validity as an assessment of vigilance. As observed in human CPTs [5,32], rodents demonstrate an intra-session vigilance decrement not due to changes in response motivation (i.e. bias) [18]. Consistent also with human attentional tasks [5], direct comparison of mouse response latencies across the 5C-CPT, the 5CSRT, and a simple single-choice (1C-) CPT revealed a significant positive relationship between attentional load and response time — latencies were slowest in the 5C-CPT (the most challenging task), intermediate in the 5CSRT, and fastest in the 1C-CPT (the least challenging task) [18]. Furthermore, the 5C-CPT and clinical CPTs appear to engage common brain regions and networks in rodents and humans, respectively, providing evidence for construct validity in the neurophysiological domain as well. Specifically, human CPT performance is closely tied with activation of a fronto-striato-parietal network [29,31,33], and indeed, lesioning the parietal cortex (PC) impairs rodent 5C-CPT performance [31]. That PC activation occurs during both target and non-target 5C-CPT trials in humans [31] but is not required for rat 5CSRT performance [34] further highlights the importance of non-target stimuli in cross-species attentional paradigms. It should be noted, however, that additional work at the receptor and/or connectome level is needed in order to definitively assert that 5C-CPT-related activation of the PC and other regions produces the same downstream effects across species [35].

The consistency of the 5C-CPT with the cognitive construct of vigilance and the cross-species convergence in underlying network activity likely contribute to the assay's considerable predictive validity. Various experimental manipulations affect 5C-CPT performance in a similar manner across species and different clinical CPTs. For example, in accordance with findings from an earlier clinical CPT [36], a reverse-translated version of the 5C-CPT identified a similar reduction in vigilance by sleep deprivation across mice and humans [37]. Meanwhile, administration of amphetamine enhanced similar measures of 5C-CPT performance in humans, mice, and rats [38–40]. This amphetamine-induced improvement of human attention replicated findings generated by the commonly used Conners’ CPT, thus demonstrating inter-paradigm consistency with a benchmark clinical assessment [41]. The reverse-translated 5C-CPT is also highly consistent with both the Conners’ CPT and the CPT-Identical Pairs (another gold-standard clinical CPT) when assessing psychiatric populations [42,43], thus providing additional psychometric validation. The 5C-CPT therefore enables more confident extrapolation of the effects of pharmacological and/or environmental manipulations on rodent task performance to human vigilance than the earlier 5CSRT and SAT.

## The 5C-CPT as an assessment of psychopathology-associated vigilance deficits

The cross-species translatability of the 5C-CPT demonstrated above has been repeatedly leveraged to assess the role of putative risk genes and dysregulated neurotransmission in the specific attentional deficits observed in ADHD, schizophrenia, and BD. For example, the 5C-CPT identified increased response perseveration (a possible analog of compulsive checking behavior in ADHD) in mice lacking functional neurokinin-1 receptor (NK1R) [44,45], a model of certain genetic ADHD patient subgroups [46,47]. These mice displayed neither inattention nor impulsivity-like behaviors, however [44,45], limiting the applicability of their 5C-CPT performance to this one aspect of the ADHD cognitive syndrome. Nevertheless, methylphenidate normalized perseveration consistent with reductions in compulsive checking in young methylphenidate-treated ADHD patients [48]. This effect provides evidence for a link between clinical efficacy of methylphenidate and status of the human NK1R analog gene, *TACR1* [45] — an important finding given the high rate of non-response to this treatment amongst ADHD patients [49].

Other research has utilized the inter-individual heterogeneity of rodent attention to assess the effects of frontline and novel ADHD medications on rats of high and low baseline inattention and impulsivity. This strategy generates cohorts of rats that model the three subtypes of adult ADHD (inattentive, hyperactive-impulsive, and combined) that demonstrate high levels of pharmacological predictive validity when assessed in the 5C-CPT. Specifically, traditional ADHD therapeutics improved accuracy and overall vigilance in ‘low-attentive' rats and normalized motoric impulsivity and/or response disinhibition (two mechanistically dissociable types of impulsive behavior; [50]) in ‘high-impulsive' rats [51]. Subsequent application of this model to the assessment of putative novel pharmacotherapies identified therapeutic potential for the catechol-*O*-methyltransferase inhibitor tolcapone, the α7 nicotinic acetylcholine receptor (α7-nAChR) agonist encenicline, and a selective dopamine D4 receptor (DRD4) agonist [52,53]. The putative clinical utility of these drugs indicated by the 5C-CPT is corroborated by other studies utilizing this task and related assessments. For example, in addition to producing similar 5C-CPT effects as traditional psychostimulant medications [38,51], tolcapone reduced false alarm rates in healthy humans with low baseline performance while simultaneously enhancing an EEG correlate of correct rejection [54]. Meanwhile, nicotine improved mouse 5C-CPT performance in an apparently α7-nAChR-dependent manner [55,56], and also improved clinical CPT performance in non-smoking adults with ADHD and low attentiveness [57]. Finally, mice with reduced DRD4 expression exhibited deficits in the 5C-CPT, driven by response disinhibition [50]. While further testing is necessary to assess the therapeutic potential of these drugs and the clinical relevance of *TACR1* polymorphisms, the 5C-CPT demonstrates considerable utility in the context of preclinical ADHD research.

Pharmacological, genetic, and developmental animal models of schizophrenia have also been assessed in the 5C-CPT. Consistent with patient CPT performance [14,58], the non-competitive *N*-methyl-D-aspartate receptor (NMDAR) antagonism rat model of schizophrenia [59] reliably demonstrates 5C-CPT deficits across assessments and laboratories [60–62]. Namely, reductions in overall vigilance and hit rate and increases in false alarm rate were observed in rats following acute MK-801 administration [62], as well as acute, subchronic, and post-subchronic (i.e. washout) phencyclidine (PCP) [60,61]. This specific pattern of reduced target detection in combination with impaired cognitive control matches that demonstrated by schizophrenia patients assessed in the human 5C-CPT [28,30], indicating consistency of the NMDAR antagonism model with the construct of schizophrenia-associated vigilance when assessed in this paradigm. Together with the predictive validity of the 5C-CPT itself, this cross-species similarity in task performance enables the use of this model to assess therapeutic potential of pro-attention drugs in the context of schizophrenia. Indeed, rescue of MK-801-induced vigilance deficits and PCP-induced disinhibition by a dopamine D1 receptor (DRD1) agonist [61,62] aligns with previous evidence that such treatment may enhance cognition in schizophrenia [63]. Other schizophrenia-related manipulations assessed in the 5C-CPT include hypoexpression of Sp4 [64] (a transcription factor linked to schizophrenia and BD; [65,66]) and developmental vitamin D deficiency [67] (a proposed mechanism for demographic correlations with schizophrenia and other psychiatric diagnoses, e.g. BD; [68]). While these manipulations did not produce attentional profiles of the same nuanced similarity to schizophrenia as NMDAR antagonism or share that model's specificity to the disease, both models effected reductions in task performance that were sensitive to either established (clozapine; [67]) or putative (glycine transporter inhibitor; [64]) schizophrenia therapeutics. While these latter findings are still preliminary, the robust cross-species translatability of the 5C-CPT strongly recommends follow-up study of these drugs and genetic/developmental risk factors.

The neural substrates of specifically BD-associated inattention were investigated by assessing patients in the 5C-CPT during manic and euthymic states alongside dopamine transporter knock-down (DAT KD) mice [31]. DAT KD mice reproduce the DAT hypoexpression profile occurring in BD [69,70], and display robust cognitive and behavioral profiles that closely align with those of BD patients [71–73]. Importantly, DAT KD behavior is normalized to various degrees by mood stabilizers [74,75], enabling preclinical evaluation of putative therapeutics [76,78]. DAT KD mice demonstrated severely impaired 5C-CPT performance characterized by a pattern of inattention, motoric impulsivity, and disinhibition highly similar to that of acutely manic BD patients tested in parallel. Euthymic BD patients performed similarly to controls, albeit with somewhat lower hit rates driven by more misses. Concurrent fMRI revealed reduced bilateral PC activation in response to non-targets in euthymic patients self-reporting high impulsivity during positive mood states. Meanwhile, mice with bilateral PC lesions demonstrated a general vigilance deficit in the 5C-CPT [31]. Interestingly, performance of the target-only 5CSRT was not affected in rats with similar PC lesions [34], and was significantly *enhanced* in DAT KD mice [31]. Taken with euthymic patients’ reduced PC activation following non-target stimuli, this differential PC involvement in 5C-CPT versus 5CSRT and similarly divergent performance of the two tasks by DAT KD mice links BD-relevant reductions in PC activation and DAT expression to inappropriate response selection to target versus non-target stimuli. These findings from the 5C-CPT therefore open the door to subsequent studies of the role of dopamine clearance in the PC versus other areas (e.g. striatum) in the cognitive and behavioral deficits observed in BD [31]. Additionally, given that dopamine clearance also occurs via various other mechanisms elsewhere in the brain, delineation of the contributing roles of other dopamine regulators beyond the DAT to BD symptomology (e.g. norepinephrine transporter, catechol-O-methyltransferase, monoamine oxidase B) will be vital moving forward [79].

In summary, the 5C-CPT is a well-validated, clinically sensitive assay that has already proven useful in: (1) delineating neural mechanisms underlying vigilance; (2) characterizing animal models of clinical populations; and (3) identifying potential therapeutics that can be tested in patients.

## The touch screen rodent continuous performance test (rCPT)

The rCPT (Figure 1) is a recent addition to the field of cross-species vigilance assessment. Utilizing a touchscreen interface rather than a linearly distributed array of stimulus delivery/response apertures, this test improves upon the 5C-CPT by enabling delivery of nuanced, centrally displayed (i.e. non-spatial) stimuli more consistent with those utilized in traditional clinical CPTs [80]. Clinical assessments like the Conners’ CPT utilize alphanumeric stimuli, meaning that targets and non-targets share many features and are distinguishable only by relatively subtle differences in shape. The rCPT vastly expands the possible stimuli beyond those of the 5C-CPT to include complex shapes and patterns that more closely approximate such clinical parameters. Task difficulty can also be modulated by adjusting stimulus size and contrast, introducing distractors, or otherwise degrading the stimuli. Furthermore, the rCPT removes the confound of spatially divided attention present in the 5C-CPT (again consistent with traditional clinical CPTs) by delivering stimuli and receiving responses via a single centrally localized touchscreen [80]. Importantly however, the rCPT in its standard form delivers target and non-target stimuli in a 1 : 1 ratio, making it akin to a go/no-go task [81]. In this regard, the 5C-CPT, which follows a 5 : 1 target/non-target ratio [18], maintains an advantage as it enables assessment of prepotent response inhibition [27]. The rCPT is readily adaptable however, and clinical relevance can be improved by adjusting the target/non-target ratio to 9 : 1 (like the Conner's CPT; [23]), 3 : 1, or even 1 : 3 or 1 : 5 (like the original X-CPT; [12]).

Despite its reduced capacity to assess response inhibition, the rCPT nevertheless demonstrates validity as an assay of vigilance and impulsivity/disinhibition. Lesion studies have illustrated cross-species convergence in neural substrates underlying response inhibition in the rCPT and the clinic [82,83]; namely, ablation of the anterior cingulate cortex increased false alarm rate and motoric impulsivity (i.e. premature responding) [82], consistent with clinical findings implicating this region in error detection and cognitive control [84–86]. Importantly, rat performance of the rCPT is sensitive to manipulations of the same task parameters as human CPT performance (e.g. inter-trial interval, introduction of distractors, and stimulus quality) [80]. The rCPT also identified pro-attentional and anti-impulsivity effects of various ADHD medications on wildtype mice with, respectively, low baseline vigilance and high baseline impulsivity; these effects were consistent with the ‘inverted U-shaped’ dose-response curve observed in the clinic [87,88], thus demonstrating pharmacological predictive validity. Clinically approved stimulant medications also increased hit rate and response bias in the rCPT across a cohort of rats not grouped by baseline performance (albeit while concurrently increasing disinhibition to the detriment of composite vigilance scores) [89].

As with the 5C-CPT, the construct and predictive validity of the rCPT enables its use in the context of preclinical psychiatric research. To date, three studies have utilized the rCPT to characterize the attentional profiles of neurodevelopmental models of schizophrenia and ADHD. Rats exposed to the neurotoxin methylazoxymethanol acetate (MAM) during gestation — a model of schizophrenia-relevant neurological abnormalities and behavioral and cognitive deficits [90,91] — demonstrated impaired overall vigilance in the rCPT, driven by response disinhibition and motoric impulsivity [92]. Another prenatal ‘exposure' paradigm — maternal nicotine ingestion, a risk factor for ADHD in humans [93] — delayed rCPT acquisition, reduced overall vigilance, and increased motoric impulsivity in mice [94]. Finally, a mouse model of 22q11.2 deletion syndrome — a major risk factor for schizophrenia and ADHD [95] — demonstrated a reduced hit rate and overall vigilance impairment [96]. As well as generating the first direct evidence for attentional impairment in these models, the rCPT enabled the screening of several putative pro-cognitive drugs for effectiveness in remediating these deficits. While the deficits of MAM-treated rats were resistant to pharmacological remediation [92], amphetamine succeeded in dose-dependently enhancing overall vigilance in 22q11.2-model mice [96], consistent with clinical findings [97,98]. Additionally, mice gestationally exposed to nicotine exhibited functionally altered glutamate receptors in hippocampal CA1 pyramidal neurons, in conjunction with their attentional deficit [94]. Meanwhile, the 22q11.2-model mice exhibited reduced prefrontal-hippocampal oscillatory synchrony following termination of rCPT assessment [96]. While no statistical correlations were reported between these neural measures and task performance or drug responsivity, these findings nevertheless suggest future investigation of hippocampal mechanisms underlying rCPT performance (despite the apparent non-involvement of this region in healthy human CPT; [99]). Although more work is needed to validate this task to the same level of rigor as the 5C-CPT, the design of the rCPT represents the potential for preclinical vigilance testing of greater translational relevance than that afforded by its predecessors.

## Conclusions

Here, we provided a brief history of the use of rodent behavioral tests developed to explicitly assess sustained attention. It is important to note that while all behavioral tasks require attention to a certain degree (with claims of direct attentional assessment in some tasks), we argue that, without outcome measures specifically designed to assay attention or validation for human testing, the translatability of their findings to human-relevant attention must be questioned. Even the well-validated 5C-CPT and rCPT have limitations. For example, the training time required often precludes the testing of juveniles and adolescents, thus limiting the ability to assess animal models relevant to childhood ADHD, juvenile onset disorders, and prodromal and first-break schizophrenia. Some protocols (e.g. home-cage-based autoshaping paradigms or olfactory-based assessments; [100–102]) may expedite the training process, but require further development. Another limitation is that of interpretation — response bias does not necessarily provide a full picture of rodents’ motivation. For example, without the challenge of target localization present in the 5C-CPT, animals performing the rCPT may come to discount the occasional punishment following a false alarm if non-target stimuli are relatively uncommon — for example, one in every six trials as in Conner's CPT — and simply respond in the single stimulus presentation zone indiscriminately for an 83% chance of reward. Such a strategy would result in a high response bias, without a given animal actually attending to stimulus identity. Bias measures do however offer insight into disease manipulation- and/or drug-induced changes in response rates that are not accompanied by changes in performance. Regardless, such an issue may be addressed by decreasing the ratio of target to non-target stimuli (while still allowing the development of a prepotent target response), and/or by incorporating other tasks to directly assess animals’ motivation to minimize punishment.

The 5C-CPT and rCPT can be readily applied to the study of neuropsychiatric conditions beyond those described above. For example, the underlying mechanisms of Alzheimer's disease and delirium, in which inattention is consistently identified by clinical CPTs [103,104] and related paradigms [105,106], may be elucidated via assessment of animal models of these disorders (e.g. [107,108]). Additionally, the reverse-translational approaches described herein can also be applied to other aspects of attention, such as attentional cueing using a Posner-style task [109]. As in humans, this task cues rodents to one location or another ahead of stimulus delivery and records differences in reaction time following valid versus invalid cues, thus enabling more finite forms of attention to be assessed across species. Although further validation of this latest task is required, reverse-translational approaches such as these yield clinically relevant outcomes.

In summary, current cross-species attention assessments owe a great deal to earlier operant tasks, such as the 5CSRT and SAT; however, while these older tasks remain useful, the data generated by the more recent CPT-like paradigms appear to have greater translatability to humans. The 5C-CPT is well-validated as a cross-species vigilance assay, and is also clinically sensitive and compatible with EEG and fMRI. The rCPT, though yet to be validated in this depth, offers a flexibility in testing that will enable assessment of more aspects of attention. The preliminary neurophysiological construct validity of the 5C-CPT and rCPT represents great promise for future rodent vigilance testing, with the potential for incorporating modern neuroscientific techniques to determine underlying circuit networks and neurotransmitter activity. These tasks, in combination with improved psychiatry-relevant manipulations in rodents, will ultimately facilitate discovery of targeted therapeutics for attentional enhancement.

## Summary

- Attentional dysfunction is a critical aspect of many psychiatric conditions, yet few pharmacotherapies exist for the specific remediation of such deficits.
- Cross-species translatable cognitive assays enable study of the neural substrates underlying attention in humans and rodents at a higher level of resolution than is possible with clinical techniques.
- The 5-choice continuous performance test (5C-CPT) and rodent continuous performance test (rCPT) measure sustained attention in rodents in a manner consistent with benchmark clinical assessments, and engage similar brain regions across species.
- The 5C-CPT and rCPT are sensitive to similar pharmacological and parametric manipulations as analogous clinical tasks, and animal models of psychiatric disease tested in these paradigms demonstrate attentional deficits consistent with those observed in clinical populations.
- The 5C-CPT and rCPT enable identification of novel therapeutic targets and pharmacological interventions for the remediation of attentional deficits.

## Open Access

Open access for this article was enabled through a transformative open access agreement between Portland Press and the University of California.

## Abbreviations

5C-CPT: 5-choice continuous performance test
5CSRT: 5-choice serial reaction task
ADHD: attention deficit hyperactivity disorder
BD: bipolar disorder
CPT: continuous performance test
DAT KD: dopamine transporter knockdown
DRD1: dopamine receptor D1
DRD4: dopamine receptor D4
EEG: electroencephalography
fMRI: functional magnetic resonance imaging
MAM: methylazoxymethanol acetate
NK1R: neurokinin 1 receptor
NMDAR: *N*-methyl-D-aspartate receptor
PC: parietal cortex
PCP: phencyclidine
rCPT: rodent continuous performance test
SAT: sustained attention task
SDT: signal detection theory
α7-nAChR: α7-nicotinic acetylcholine receptor
